# Knowledge and Misinformation About Breast Cancer Risk Factors, Symptoms, and Prevention Among Healthy and Affected Women: A Study on 2375 Italian Participants

**DOI:** 10.3390/healthcare12212126

**Published:** 2024-10-24

**Authors:** Luana Conte, Roberto Lupo, Alessia Lezzi, Matilde Mieli, Stefano Botti, Ivan Rubbi, Maicol Carvello, Francesco Giotta, Raffaella Massafra, Elsa Vitale, Giorgio De Nunzio

**Affiliations:** 1 Department of Physics and Chemistry, University of Palermo, 90128 Palermo, Italy; 2 Advanced Data Analysis in Medicine (ADAM), Laboratory of Interdisciplinary Research Applied to Medicine (DReAM), University of Salento, ASL (Local Health Authority), 73100 Lecce, Italy; giorgio.denunzio@unisalento.it; 3 “San Giuseppe da Copertino” Hospital, ASL (Local Health Authority), 73100 Lecce, Italy; robertolupo_2015@libero.it; 4 Department of Palliative Care, National Cancer Association (ANT) Italia Foundation ONLUS, 73100 Lecce, Italy; 1alessia.lezzi@gmail.com; 5 C.R.A.P. Comunità Riabilitativa Assistenziale Psichiatrica, 73056 Taurisano, Italy; matildemieli99@gmail.com; 6 Hematology Unit, Azienda USL-IRCCS, 42122 Reggio Emilia, Italy; stefano.botti@ausl.re.it; 7 School of Nursing, University of Bologna, 48018 Faenza, Italy; ivan.rubbi@auslromagna.it; 8 Community Hospital, ASL (Local Health Authority), 48100 Romagna, Italy; maicol.carvello@gmail.com; 9 Medical Oncology Unit, IRCCS Istituto Tumori “Giovanni Paolo II”, 70124 Bari, Italy; f.giotta@oncologico.bari.it; 10 Laboratory of Biostatistics and Bioinformatics, IRCCS Istituto Tumori “Giovanni Paolo II”, 70124 Bari, Italy; r.massafra@oncologico.bari.it; 11 Scientific Directorate, IRCCS Istituto Tumori “Giovanni Paolo II”, 70124 Bari, Italy; e.vitale@oncologico.bari.it; 12 Laboratory of Biomedical Physics and Environment, Department of Mathematics and Physics “E. De Giorgi”, University of Salento, 73100 Lecce, Italy

**Keywords:** breast cancer, prevention, knowledge, misinformation, education

## Abstract

Background: Breast cancer is the most common cancer among women worldwide and remains the leading cause of death among Italian women. Despite increased breast cancer awareness and improved diagnostic techniques, mortality rates remain high globally. In Italy, despite the availability of screening programs by the National Health System (NHS) for all Italian women aged 50–69 every two years, the participation rate remains relatively low. The low uptake of screening may be attributed to a lack of general cancer knowledge among women, including awareness of risk factors, symptoms, and prevention measures. This study investigates the knowledge and misinformation in a population of Italian women regarding breast cancer risk factors, symptoms, and prevention. Methods: From March 2021 to January 2022, we conducted a survey targeting the female population in Italy, with a total of 2375 participants willingly participating in the study. To investigate factors linked to variations in attitudes toward breast cancer, the participants were categorized into two groups: the general population (Group A, *n* = 2235) and women who have had or currently have breast cancer (Group B, *n* = 140). Statistically significant differences were identified between these two groups. Results: The findings revealed considerable confusion regarding both the symptoms and causes associated with cancer, as well as prevention measures. This confusion was particularly prominent among women in the general population and those with lower levels of education. Conclusions: Given these insights, it remains crucial to promote accurate health information concerning risk factors, symptoms, and prevention strategies related to this devastating disease, emphasizing the ongoing importance of disseminating correct health information.

## 1. Introduction

Breast cancer represents the most common form of cancer worldwide [1,2] with 55,900 new diagnoses estimated in 2023 and 15,500 deaths recorded in Italy in 2022 [3]. Despite increased awareness and public attention, breast cancer mortality rates remain high globally and continue to be the leading cause of death in Italy [3]. Improved diagnostic techniques have enabled early detection, allowing for the identification of small tumors that may not be easily detectable. Early intervention is crucial in planning a treatment and/or surgery for neoplasms at an early stage [2]. Thus, an early diagnosis remains the most vital factor in improving the patient prognosis, with prevention and adherence to screening programs being the most effective strategies. Mammography screening is a periodic secondary prevention measure aimed at women, enabling the earliest possible diagnosis of breast cancer. This approach facilitates less aggressive and more effective treatments, ultimately reducing mortality from this devastating disease [3]. In Italy, in line with the prevention guidelines, mammography screenings are provided free of charge every two years to women aged 50–69 [3]. It has been observed that mortality reduction for women in this age group is estimated at 23% for both adherent and non-adherent individuals, while women adherent to screening experience a 40% reduction. However, this opportunity is not always utilized, and significant geographical disparities have been observed in terms of screening program implementation, breast cancer incidence, and survival rates [4,5]. Lack of knowledge regarding risk factors, symptoms, and prevention strategies for cancer may underlie the low adherence to screening programs. For instance, results from a European and Italian survey indicate inadequate awareness in Italy regarding the conscious approach to screening examinations [6]. Extensive studies conducted in recent years have revealed that 20–30% of newly diagnosed breast cancer cases are associated with various risk factors that actively initiate or modify the neoplastic transformation of breast cells [3,7,8,9,10,11,12]. Age at first birth [3], genetic factors, endocrine influences, diet, environmental factors, lifestyle, and previous breast conditions are all linked to the risk of developing breast cancer [13]. Notably, advancing age increases the risk due to endocrine proliferative stimuli and the accumulation of genetic transcription errors in cellular DNA [3]. Hormonal factors such as early menarche, late menopause, and hormone therapy use play a role [9]. Genetic predisposition [14], including alterations in the BRCA-1 and BRCA-2 genes, carries lifetime risks of 65% and 40% for developing breast cancer, respectively [3]. Lifestyle parameters affected by factors such as obesity [15,16], diet [17,18,19], excessive alcohol consumption, smoking, stress [8], and pollution [7] contribute to the risk as well [20]. Therefore, mammography, and other medical imaging [21,22,23], plays a crucial role in cases where early symptoms of breast cancer, such as palpable lumps, are present. Another prevention method that is highly debated but strongly recommended is breast self-examination (BSE) [24,25]. BSE involves the periodic self-palpation of the breasts by women to identify any changes that should be reported to their healthcare provider. While self-examination alone is not sufficient in an age where mammography and breast ultrasound can detect tumors as small as a few millimeters, it should be noted that when performed correctly and regularly, this technique can help reduce the risk of diagnosing advanced breast cancer. Moreover, self-examination is a convenient and cost-effective method that women can start from the age of 20 [26]. Educating women about the benefits of self-examination is crucial, as it is the easiest and most accessible way to detect breast cancer at an early stage [26].

Therefore, prevention is essential, but equally important is knowledge about cancer. The more people are aware of the risk factors and symptoms of breast cancer, the more likely they are to approach screening positively. Against this backdrop, the objective of this study is to investigate the knowledge and misconceptions regarding breast cancer among Italian women, focusing on risk factors, symptoms, and prevention.

## 2. Methods

### 2.1. General Study Details

Between March 2021 and January 2022, a survey was undertaken targeting the female population in Italy. A total of 2375 participants voluntarily participated in the study by completing an anonymous questionnaire. The survey, distributed on a voluntary basis, was conducted exclusively among Italian women aged 20 to 69 who provided informed consent. As this study was survey-based and the questionnaire was sent to women who voluntarily responded, approval from the institutional ethics committee was not sought.

### 2.2. Participants

Exclusion criteria involved non-compliance with the age range or lacking Italian citizenship. The questionnaire was digitally implemented using a predefined form on the Google Forms platform, and the study employed electronic means for disseminating the questionnaire. Outreach efforts involved contacting various Facebook groups and Instagram pages that featured computerized questionnaires.

### 2.3. Aim

The questionnaire was administered to assess the knowledge and misinformation in a population of Italian women concerning risk factors, symptoms, and the prevention of breast cancer. 

### 2.4. Study Methodology

The predesigned, not-pretested questionnaire was expected to take approximately 10 min to complete. Socio-demographic data such as the age, geographical area of residence, marital status, level of education, and employment status were collected. The administered questionnaire consisted of 43 items divided into two sections. The first section (11 items) focused on knowledge and beliefs about the causes and symptomatology of breast cancer. The second section (32 items) assessed knowledge and beliefs about breast cancer prevention. The questionnaire was sent through social networks. The sampling approach employed virtual snowball sampling until data saturation was achieved. An unanswered questionnaire is attached as Appendix A. 

### 2.5. Ethical Considerations

The ethical considerations of the study were explicitly outlined during the presentation of the questionnaire. The design of the questionnaire adhered to the principles established by the Italian data protection authority (DPA). The study was approved by the Bioethical Committee of IRCCS, Bari, under protocol number 1695/CEL dated 10 June 2024, conducted according to ethical guidelines established by the Declaration of Helsinki and other guidelines like Good Clinical Practice Guidelines. Emphasis was placed on the voluntary nature of participation, with participants retaining the right to decline involvement in the protocol at any point. Individuals expressing interest in participating were provided with an informed consent form that reiterated the voluntary nature of participation and underscored the confidentiality and anonymity of the shared information. To uphold anonymity, the responses of participants were deidentified.

### 2.6. Definition

The term “general population” refers to the entire set of individuals or people within a specified geographic area or community who share common characteristics or attributes. This group typically represents a broad and diverse cross-section of society, encompassing people of various demographics, ages, backgrounds, and lifestyles. In research and surveys, the general population serves as the target group from which researchers draw samples to make inferences about broader trends, attitudes, behaviors, or characteristics. It contrasts with specific subpopulations, which may be defined by certain criteria such as age, gender, ethnicity, or other factors.

“Women with a history of breast cancer” refers to individuals of the female gender who have been previously diagnosed and treated for breast cancer. This term encompasses women who have undergone medical interventions, such as surgery, radiation therapy, chemotherapy, or a combination of these, to address the presence of breast cancer in their medical history. The phrase is often used in medical and research contexts to categorize a specific subgroup of individuals for the purpose of studying or addressing issues related to breast cancer survivorship, treatment outcomes, and long-term health considerations.

### 2.7. Statistics

Descriptive statistics were employed to report the questionnaire responses from all participants. To pinpoint items linked to variations in breast cancer-related behavior, the subjects were categorized into two groups: the general population (Group A, *n* = 2235) and women with a history of breast cancer (Group B, *n* = 140). For each question, respondents were further stratified by the age, educational level, geographic area, and marital status, as applicable. Continuous variables were summarized using the mean and standard deviation (SD), while categorical variables were presented with frequencies and percentages. Group differences were assessed using the Mann–Whitney U-test, considering a *p*-value < 0.05 as statistically significant. All statistical analyses, encompassing both qualitative and quantitative variables, were performed using MATLAB software (R2023b).

## 3. Results

**Figure 1:**
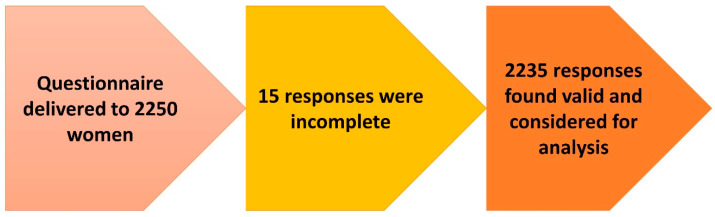
Flow diagram for questionnaire participant selection.

The questionnaire was delivered to 2250 eligible women. Complete responses were received from a total of 2235 (99%) and included in the final analysis (Figure 1).

Baseline characteristics were assessed for all participants, and the data are summarized in Table 1. To explore potential differences in women’s information, knowledge, and beliefs regarding breast cancer, the respondents were divided into two groups: Group A, which includes women who have not been diagnosed with breast cancer (94%, *n* = 2235, referred to as the “general population”), and Group B, which includes women who have already been diagnosed with breast cancer (6%, *n* = 140).

Section 1 of Table 2 specifically investigates respondents’ knowledge of causes and symptomatology associated with cancer. As expected, women in the general population report were less informed than women with prior cancer (56% vs. “93%” for the cumulated “very” and “fairly” answers, respectively), *p* < 0.05. It would also appear that this lack of knowledge on the topic is correlated with age: almost half (48%) of the women in the general population who say they are poorly informed are mainly in the 20–30 age group. The level of education also seems to affect this trend as women in Group A with no or a very low level of education admit to knowing little about the disease.

Going into detail about the knowledge they possessed, respondents were asked whether they considered endocrine factors, previous breast disease, diet, pollution, and psychological stress to be possible causes of breast cancer occurrence. Although it is now known that higher-than-normal levels of sex hormones, estrogens and androgens, can promote the occurrence of heredo-familial cancers such as breast or prostate cancer, there are many women who deny this relationship. Surprisingly, it is mostly women with previous cancer compared to others who exclude endocrine involvement among the causes of cancer (29% vs. 17%, respectively), *p* < 0.001. The level of education also affects the responses, considering that half of the women in the general population (50%) who deny such endocrine involvement have no level of education.

Breast cancer is believed to have a strong familial predisposition. Of course, not all breast pathologies lead to heritable cancers, and not all breast pathologies affect the occurrence of cancer in the same woman who had previous pathologies. When asked whether previous breast disease affected the occurrence of breast cancer, women in both groups gave affirmative responses (95% vs. 88%: Group A and Group B, respectively), *p* < 0.01. Again, the level of education affects the responses, given that women who tended to deny a relationship between previous breast disease and cancer either lacked education (50%) or possessed only a junior high school diploma (57% and 67%: Group A and Group B, respectively).

Nutrition is another highly debated factor between the two groups of respondents (*p* < 0.001). The combination of diet and cancer occurrence is often the focus of media attention from doctors and nutritionists. Incredibly, less than half (49%) of women in the general population and only 67% of women with prior cancer know the importance of diet in cancer. Especially young women under the age of 30 (57%) and with low levels of schooling (67%) deny this relationship.

Another sustained cause of cancer involves pollution and environmental factors. Although there are many people who consider pollution to be among the leading causes of cancer, in fact, at present, this association is highly debated in the scientific field. Women with previous cancer more frequently replied “yes”, if compared with Group B (71% vs. 48%).

Lastly, there is psychological stress. The majority of women with previous cancer (71%) consider psychological stress to be a key factor in cancer occurrence (*p* = 0.01).

The following questions address knowledge about breast cancer symptomatology; specifically, women were asked whether breast pain (mastodynia), the presence of palpable lumps, breast shape/size change, and nipple alteration/secretion were symptoms associated with cancer.

Mastodynia is not one of the symptoms found in this disease unless tissue inflammation is also present (33), which occurs in only 5% of cases (34). Women in the general population prove to be more uninformed about this aspect, collecting 59% of “yes” responses in contrast to 33% of affirmative responses from women with previous cancer. The majority of “yes” votes in Group A were from women under 30 years of age and without any level of education (75%), again demonstrating that the age and level of education affect cancer knowledge. In contrast to breast pain, the presence of a palpable lump may instead be an indication of breast cancer. Women with a history of previous cancer, understandably, exhibit greater awareness of this condition, with 83% of responses indicating familiarity. However, it is more concerning that in the remaining 17% of cases, respondents do not possess this knowledge. The age and level of education correlate with “no,” garnering more very young women under the age of 30 (38%) and with low levels of schooling (100%). Interestingly, the majority of women who deny the presence of a palpable lump as the start of breast cancer are from the south and the islands.

Both groups at 71% count change in breast shape and size and nipple alteration/secretion among the possible symptoms of breast cancer. These are not symptoms specifically attributable to breast cancer, although these phenomena occur more often in older patients. Nevertheless, all women under the age of 30 (100%) considered nipple secretion to be a present symptom.

In Section 2, possible misinformation was also investigated in the area of prevention. Women were asked whether they were well informed and whether they thought it would be useful to have some of the most common clinical checkups, such as clinical palpation, ultrasound, mammography, and other tests such as blood tests and diagnostic imaging. Although almost all women knew the meaning of prevention—understood as the prevention of risk factors and an early diagnosis—it is serious to note that nearly half (47%) of the women in the general population said that they knew little or nothing at all about prevention itself, and among them, all have no level of education.

Clinical palpation, mammography, and ultrasound were considered useful as screening tests by almost all women. Schooling proves to be a crucial factor for misinformation in this area, considering that 67% of women with prior cancer and 50% of women in the general population who did not find clinical palpation and ultrasound useful possessed a primary school diploma. Surprisingly, a notable number of women are unfamiliar with the concept of a mammogram (*n* = 131, 7% in Group A and 22.2% in Group B).

In Italy, mammography is indicated in women 40 years of age and older and is offered free of charge by the National Health System (NHS) to women over 50 and up to 69 years of age. It is not normally indicated under 30 years of age. The responses were very different in the two groups (A and B) and the difference is statistically significant (*p* < 0.05). Incredibly, only 31% of women with cancer thought it was correct to have mammography in the 40–50 range and even worse, no women with previous cancer (0%) thought it was correct to have this examination in the 50–69 range, the only period covered by free screening in Italy.

Magnetic Resonance Imaging (MRI) and Computed Tomography (CT) are normally never prescribed as screening tests, except in cases of young women with pronounced genetic familiarity and cases of known staging, respectively. The standard goal for breast cancer diagnoses is certainly biopsy, but even this is not to be counted among the screening tests for preventive purposes. Still, more than 20% in both groups considered these tests useful, although many of these respondents did not have any level of education.

Blood tests are also not useful for breast cancer diagnoses. Yet, strangely, 34% of women with cancer considered them useful, and this percentage rises in the group of women in the general population (52%) (*p* < 0.001). Again, most of the women under the age of 30 or without any level of education thought that blood tests were useful. Similarly, it concerns the intervention of an oncologist. The oncologist is the reference figure in the case of established diagnoses, and normally does not intervene in the screening phase. In contrast, 35% of women with cancer considered that talking to the oncologist can be useful, a percentage that rises to 45% in the case of women in the general population (*p* < 0.01).

In the remaining part of the questionnaire, the survey explores knowledge on the topic of self-palpation. Almost all women in both groups said that they had heard of self-palpation (97% in Group A and 98% in Group B) and most of them knew the meaning of the term (82% in Group A and 87% in Group B), with the exception of a group of very young women under 30 or those with no or low levels of schooling. The percentages, however, drop dramatically when it comes to how much self-examination can help in cancer prevention: surprisingly, less than half (41%) of women in Group A and just over half (56%) of women in Group B strongly agreed that it is a valuable aid, and the women who describe themselves as uncertain tend to be those with low levels of education. While self-palpation was also considered an important tool for periodic monitoring even following breast examination and/or mammography, neither group of women responded unanimously, with some even expressing uncertainty about this, especially among women with previous cancer (*p* < 0.05). There is disagreement regarding the effectiveness of self-palpation for the reduction in mortality: self-palpation is a self-diagnosis tool that could aid in detecting palpable nodules, thus enabling early intervention and diagnoses. Regular self-examination, in addition, could serve to find a breast lump early, which would mean early intervention to limit the consequences of cancer. Therefore, it is crucial to diagnose the disease as early as possible to reduce mortality. Despite this well-known evidence, many women disagreed on the issue in both groups (*p* < 0.05), and many expressed uncertainty, even among the group with previous cancer (23%). A significant number of them, however, have no educational level or only a primary diploma. Among the two groups surveyed, women with cancer were in general more aware (*p* < 0.05), but a large proportion of uncertain women were nonetheless present (24% in Group A and 19% in Group B).

## 4. Discussion

The aim of the study was to investigate women’s knowledge and misinformation about breast cancer. Specifically, the information possessed by the respondents was divided into several sections. Section 1 investigated the knowledge possessed about the causes and possible signs and symptoms associated with cancer; Section 2 investigated the topic of prevention, understood as the perception of the usefulness of some common diagnostic tests such as clinical palpation, ultrasound, mammography, biopsy, and others such as blood tests and diagnostic imaging (MRI and CT) in preventing breast cancer. The knowledge regarding self-palpation as an important tool for periodic monitoring of breast changes was also investigated. In order to ascertain any difference in information possession, women were divided into two groups: Group A including women who had not been diagnosed with breast cancer and Group B including women who had already been diagnosed with breast cancer.

The analysis of the above data shows that almost half of Group A women admitted to have little knowledge about cancer, unlike women with previous cancer, who—as expected—reported being more informed, and this is in line with data obtained from a cross-sectional survey conducted in five European countries including Italy [6]. This is a significant finding that draws attention to the need to increase health information. Indeed, a great deal of misinformation has emerged regarding the role of endocrine factors, the presence of previous breast disease, nutrition, pollution, and psychological stress as possible risk factors for the onset of breast cancer [6]. Although over the past two decades the involvement of endocrine factors in the occurrence of heredo-familial cancers has been well established, and the concept of nutritional support as part of a comprehensive cancer management program has gained increasing interest [9,10,11,12,27], there were many women who denied such correlations with cancer. While diet as a modulable factor for cancer prevention is often the focus of media attention, the relationship between environmental pollution and the occurrence of breast cancer is currently highly debated in the medical–scientific arena. There were many women interviewed who supported this pairing, especially those who had the disease in the past. In fact, there are no conclusive studies, although some observations suggest a hypothetical increase in exposed women [7,16]. Psychological stress is also considered as a probable cause of cancer [14], in line with some recent studies [28,29], in which a high level of perceived stress has been shown to be among the modifiable risk factors.

Much misinformation has also been observed about the symptomatology with which the tumor might present itself, in line with other studies in the literature [30,31,32]. Generally, in its early stages, the disease does not give rise to specific symptoms. Despite this, there are a large number of women, from Group B, who found breast pain, nipple alteration/secretion, and change in shape and size to be among the detectable symptoms. In contrast to these almost always non-evident signs, the presence instead of a palpable lump may be indicative of breast cancer [19]. Even on the subject of prevention, the women interviewed admitted to have strong misinformation. A systematic review of 35 studies published between 1992 and 2017 regarding women’s knowledge about mammography found that there is a great deal of confusion regarding the age and frequency with which mammography should be performed, with a very strong underestimation [33]. This serious confusion was also confirmed in our study. Although in Italy mammography is indicated in women over 40 and is offered free of charge by the National Health System (NHS) to women over 50 and up to 69 years of age, there are still very few women who are aware of it and, even stranger, only 4% of Group A and, incredibly, no woman with previous cancer considered it advisable to perform this examination in the 50–69 range, the only period covered by free screening. This figure indicates a strong gap regarding knowledge of the guidelines provided and probably lack of adherence to the screening offered by the NHS in the specific age range. Coupled with this confusion is also an indecision regarding the frequency with which mammography is recommended. The Ministry of Health recommends mammography for women between the ages of 50 and 69, every two years [34], although some regions are testing the effectiveness of mammography in a wider age range [13,35,36]. On the other hand, it is not recommended under the age of 40, as many of the women in the general population have felt.

Similarly to mammography, clinical palpation and ultrasound are also perceived as useful screening tests by the majority of women in both groups. Although ultrasonography is not generally recommended as a screening test in place of or in addition to mammography, and MRI, CT, and blood tests are not screening tools but are recommended for possible post-diagnosis follow-up [37], many women in both groups considered the usefulness of these tests in the screening setting. The oncologist is also the reference figure in the case of established diagnoses, but is still perceived as supporting prevention. In the remaining part of the questionnaire, knowledge toward a breast self-diagnosis was investigated. Breast palpation is recommended as early as age 20 and can be performed by clinical examination or breast self-examination and allows each woman to get to know her breasts and appreciate any changes that might occur in the interval between one examination and the next [38]. Incredibly, only 1% of women in both groups strongly agree about its preventive role, with even a high percentage of women strongly disagreeing about its usefulness in prevention. This is an important finding that again suggests to us the need for further appropriate educational and informational interventions; indeed, it is likely that a knowledge gap exists about breast cancer risk and in subsequent screening and prevention recommendations [39,40].

One of the key contributors to the observed misinformation and knowledge gaps appears to be the level of education among participants. Women with lower educational attainment, particularly those without a high school diploma, were less likely to possess accurate knowledge about breast cancer risk factors, symptoms, and prevention strategies. This suggests that education plays a critical role in shaping health literacy, as those with limited access to formal education may be less equipped to navigate complex health information or engage with preventive healthcare services. Additionally, the influence of media, particularly social media, should not be underestimated. The widespread availability of unverified information can lead to misconceptions, such as the belief that diagnostic tools like blood tests or MRI scans can prevent breast cancer. Finally, the lack of direct experience with breast cancer, either personally or within one’s social circle, may result in lower awareness of key preventive measures. Women who have not had a direct encounter with the disease may be less motivated to seek out accurate information, leaving them vulnerable to misinformation.

Based on the findings of this study, several actionable recommendations for public health interventions can be made. Firstly, targeted educational programs should be developed to address the significant knowledge gaps identified, particularly among younger women and those with lower educational attainment. These programs could be implemented in schools, community centers, and healthcare settings, focusing on breast cancer risk factors, symptoms, and the importance of regular screening. Secondly, given the widespread misinformation observed, public health authorities should invest in media campaigns that utilize both traditional and social media platforms to disseminate accurate, evidence-based information on breast cancer prevention. These campaigns should emphasize the importance of mammography in the recommended age range and breast self-examinations and clarify the roles of diagnostic tools like MRI and blood tests. Lastly, efforts should be made to improve access to screening services, particularly in underserved regions. Increasing awareness of the free mammography services provided by the National Health System for women aged 50–69, along with ensuring that healthcare providers actively encourage participation, could help bridge the gap in screening adherence.

The results of the study must be considered taking into account some limitations. First of all, a significant limitation of this study is the discrepancy in sample size between the two groups. The group of women with a history of breast cancer was considerably smaller than the general population group (approximately 1/16th the size). This discrepancy could have impacted the statistical power of the analyses, especially regarding the detection of significant differences between groups. The smaller sample size in the breast cancer group may have limited the generalizability of the findings and could have skewed the significance testing in some cases. While this imbalance was unavoidable due to the relative rarity of breast cancer in comparison to the general population, it is important to interpret the results with caution, particularly in terms of statistical significance. In addition, the reference sample consisted mainly of young women under 30 years of age, and only 338 women (14%) were in the 50–69 age group, potentially leading to an underestimation of this demographic segment. This limitation is surely related to the mode of administration through the telematic medium, which is probably used more by younger women. Another limitation mainly concerns the choice of the electronic dissemination of the questionnaire that may have partially excluded those who had little computer background. Possible information bias may be due to a reluctant attitude to declare and therefore admit a lack of knowledge of the phenomenon. Women who are more comfortable using digital tools or more interested in the topic of breast cancer may have been more likely to participate, which could result in an overrepresentation of certain groups. Consequently, the findings may not fully reflect the broader population, particularly those with limited internet access or digital literacy. Furthermore, self-reported data may be subject to social desirability bias, where respondents could have overestimated their knowledge or behaviors related to breast cancer prevention and symptoms, affecting the accuracy of the findings.

Another limitation of this study is that the questionnaire used has not yet undergone formal validation. While the instrument was carefully designed to address key topics related to breast cancer awareness, we acknowledge the importance of conducting a validation study to ensure its reliability and accuracy. We plan to carry out this validation in future research.

## 5. Conclusions

The results obtained, in the literature, demonstrate a lack of knowledge regarding each section provided in the questionnaire, confirming previous studies. In fact, a great deal of confusion has emerged about both the symptomatology and causes associated with cancer, but also about prevention, and this confusion is present especially among women in the general population and women with low levels of education. The great misinformation about cancer and the risk factors and symptomatology associated with it highlights an important need to provide women with more information to ensure better knowledge on the topic. There is definitely a need for better dissemination and implementation regarding the benefits of preventive practices, based on evidence, in order to increase women’s confidence in prevention pathways and consequently ensure as early a diagnosis as possible. In fact, on the subject of prevention, it should be considered that despite the fact that nowadays there is greater awareness and better management of screening programs, the number of women adhering to them is still small. Low participation in screening [41], in fact, can be attributed to low public awareness and/or numerous social, psychological barriers and social factors. Knowing the risk factors associated with cancer is also an important means for all those women who have no signs and symptoms of disease. Therefore, it is essential to promote awareness of risk factors and increased participation in mammography practice among women of a screening age. Therefore, it would be appropriate to increase the information possessed by these women and screening adherence campaigns, making use of the centers specialized in the early diagnosis and treatment of breast cancer, such as the Breast Units in the territory. There are at least 200 Breast Units and, according to the State–Regions Conference [42], they prove to be useful not only in the case of patients with cancer, but also in the case of healthy women without family history for whom it is useful to ensure prevention and early diagnoses, encouraging correct lifestyles and carrying out training activities, allowing breast examinations through which, if necessary, diagnostic tests can be accessed. In light of these data, it therefore remains of utmost importance to promote correct health information on the subject of risk factors, symptomatology, and prevention associated with this terrible disease, which is still a major cause of suffering and premature mortality in women worldwide.

## Figures and Tables

**Table 1:** Baseline characteristics and the questionnaire items of all respondents. Section 2 of the Questionnaire is related to screening adhesion for all women.

Baseline Characteristics	N (=2375)	%
Age (y)		
20–29	1076	45
30–39	544	23
40–49	417	18
50–59	249	10
60–69	89	4
Geographic Area		
North	607	26
Center	512	22
South/Islands	1256	53
Marital status		
Married	944	40
Divorced	81	3
Maiden	1280	54
Separate	46	2
Widow	24	1
Education level		
Degree	954	40
High school graduation	1197	50
Junior high school diploma	202	9
Primary school	17	1
None	5	<1
Employment status		
Craftsman	254	11
Public Administration	624	26
Services/Tertiary	356	15
Student	746	31
Retired	50	2
Unemployed	345	15

**Table 2:** Questionnaire responses of the adult female population among subjects who had not been diagnosed with breast cancer (Group A, *n* = 2235) and subjects who had already been diagnosed with breast cancer (Group B, *n* = 140). Differences in response between the two Groups were assessed. A *p*-value <0.05 was considered statistically significant (* *p* < 0.05; ** *p* < 0.01; *** *p* < 0.001).

Questionnaire Items	Group A Women in the General Population (*n* = 2235) N (%)	Group B Women with Cancer (*n* = 140) N (%)	*p*-Value, *Z*
**SECTION 1: Knowledge and beliefs about the causes and symptomatology of breast cancer**			
Q1. Do you think you are well informed about breast cancer?			
Very	132 (6%)	52 (37%)	<0.05 *, −0.316
Quite	1118 (50%)	79 (56%)	
Little	898 (40%)	9 (6%)	
Not at all	87 (4%)	0	
Q2. Do you think the cause of breast cancer is endocrine?			
No	372 (17%)	40 (29%)	<0.001 ***, −3615
Yes	1863 (83%)	100 (71%)	
Q3. Do you think a cause of breast cancer may be previous breast disease?			
No	122 (5%)	17 (12%)	<0.01 **, −3268
Yes	2213 (95%)	123 (88%)	
Q4. Do you think a cause of breast cancer may be food?			
No	1136 (51%)	46 (33%)	<0.001 ***, −3688
Yes	1099 (49%)	94 (67%)	
Q5. Do you think a cause of breast cancer may be environmental factors and pollution?			
No	553 (25%)	27 (19%)	0.15, −1458
Yes	1682 (75%)	113 (81%)	
Q6. Do you think a cause of breast cancer may be psychological stress?			
No	1153 (52%)	41 (29%)	0.01 **, −5119
Yes	1082 (48%)	99 (71%)	
Q7. Do you think breast pain may be a symptom of cancer?			
No	836 (37%)	93 (66%)	0.19, −1811
Yes	1319 (59%)	46 (33%)	
I don’t know	80 (4%)	1 (1%)	
Q8. Do you think the presence of a palpable nodule may be a symptom of the tumor?			
No	713 (32%)	23 (16%)	<0.01, −4016
Yes	1452 (65%)	116 (83%)	
I don’t know	70 (3%)	1 (1%)	
Q9. Do you think that the change in breast shape and size may be a symptom of cancer?			
No	565 (25%)	38 (27%)	<0.05 *, −0.306
Yes	1579 (71%)	100 (71%)	
I don’t know	91 (4%)	2 (1%)	
Q10. Do you think nipple discharge may be a symptom of the tumor?			
No	29 (38%)	39 (28%)	0.04, −1990
Yes	45 (59%)	99 (71%)	
I don’t know	2 (3%)	2 (1%)	
Q11. Do you think that nipple alteration may be a symptom of the tumor?			
No	597 (27%)	39 (28%)	0.19, −1024
Yes	1548 (69%)	99 (71%)	
I don’t know	90 (4%)	2 (1%)	
**SECTION 2: Knowledge and beliefs about early detection**			
Q12. Do you think you are well informed about breast cancer prevention?			
A lot	123 (6%)	43 (31%)	<0.05 *, −0.516
Quite	1062 (48%)	82 (59%)	
Little	914 (41%)	12 (9%)	
Not at all	136 (6%)	3 (2%)	
Q13. What does prevention mean to you?			
Carry out periodic checks	20 (1%)	0	0.20, −1926
Prevention of risk factors and early diagnosis	2110 (94%)	128 (91%)	
Prevention of complications	80 (4%)	11 (8%)	
I don’t know	25 (1%)	1 (1%)	
Q14. If a lump is detected, will treatment be more effective?			
Strongly agree	670 (30%)	64 (46%)	<0.05 *, −0.499
Agreed	920 (41%)	45 (32%)	
In disagreement	94 (4%)	3 (2%)	
Strongly disagree	18 (1%)	1 (1%)	
Uncertain	533 (24%)	27 (19%)	
Q15. Do you consider mammography useful as an act of early detection?			
No	26 (1%)	3 (2%)	0.15, −1124
Yes	2209 (99%)	137 (98%)	
Q16. What does mammography mean to you?			
Breast self-examination and self-palpation	28 (1%)	0	0.103, −1926
Ultrasound	1 (0%)	0	
Oncologist’s physical examination	110 (5%)	1 (1%)	
Radiological examination of the breast	2075 (93%)	139 (99%)	
I don’t know	21 (1%)	21 (1%)	
Q17. At what age do you think mammography is recommended?			
<20 years old	37 (2%)	3 (2%)	0.05 *, −1972
20–30	479 (21%)	27 (19%)	
30–40	833 (37%)	65 (46%)	
40–50	786 (35%)	44 (31%)	
50–60	80 (4%)	0	
60–70	2 (0%)	0	
I don’t know	18 (1%)	1 (1%)	
Q18. How often do you think mammography is recommended?			
Based on age/familiarity	7 (0%)	2 (1%)	0.15, −1334
More than once a year	317 (14%)	11 (8%)	
Once a year	1581 (71%)	108 (77%)	
Every two years	316 (14%)	19 (14%)	
I don’t know	14 (1%)	0	
Q19. Do you consider clinical palpation useful as an act of early detection?			
No	131 (6%)	10 (7%)	0.15, −0.622
Yes	2104 (94%)	130 (93%)	
Q20. Do you think bilateral ultrasound is useful as an act of early detection?			
No	226 (10%)	7 (5%)	<0.05 *, −1972
Yes	2009 (90%)	133 (95%)	
Q21. Do you think Nuclear Magnetic Resonance Imaging (MRI) is useful as an act of early detection?			
No	295 (80%)	1650 (74%)	0.15, −1366
Yes	76 (20%)	585 (26%)	
Q22. Do you consider biopsy useful as an act of early detection?			
No	1501 (67%)	97 (69%)	0.15, −0.520
Yes	734 (33%)	43 (31%)	
Q23. Do you think Computed Tomography (CT) is useful as an act of early detection?			
No	1699 (76%)	111 (79%)	0.15, −0.881
Yes	536 (24%)	29 (21%)	
Q24. Do you consider blood tests useful as an act of early detection?			
No	1063 (48%)	92 (66%)	<0.001 ***, −4168
Yes	1172 (52%)	48 (34%)	
Q25. Do you consider the interview with the oncologist useful as an act of early detection?			
No	1226 (55%)	92 (66%)	<0.01 **, −2508
Yes	1009 (45%)	48 (34%)	
Q26. Have you ever heard of self-examination?			
No	65 (3%)	3 (2%)	0.66, −0.527
Yes	2170 (97%)	137 (98%)	
Q27. In your opinion, what does self-examination consist of?			
Breast self-examination	1840 (82%)	122 (87%)	0.11, 851
Clinical examination of the breast (search for visible and/or palpatory findings at the breast and surrounding areas, e.g., lymphatic drainage areas, axilla, neck)	337 (15%)	17 (12%)	
Breast radiological examination (mammography, ultrasound, MRI, biopsy, chest X-Ray, scintigraphy, CT scan, PET/CT, chest X-Ray)	24 (1%)	0	
I don’t know	34 (2%)	1 (1%)	
Q28. Does self-examination help in breast cancer prevention?			
Strongly agree	21 (1%)	1 (1%)	<0.05 *, −1885
Agreed	122 (5%)	11 (8%)	
In disagreement	1054 (47%)	60 (43%)	
Strongly disagree	535 (24%)	43 (31%)	
Uncertain	503 (12%)	25 (18%)	
Q29. Is self-palpation not necessary if a breast examination is performed?			
Strongly agree	2189 (98%)	136 (97%)	0.53, −0.566
Agreed	46 (2%)	4 (3%)	
In disagreement	0	0	
Strongly disagree	0	0	
Uncertain	0	0	
Q30. Is self-palpation not necessary if I perform periodic mammography?			
Strongly agree	19 (1%)	2 (1%)	<0.05 *, −1652
Agreed	143 (6%)	7 (5%)	
In disagreement	1067 (48%)	64 (46%)	
Strongly disagree	492 (22%)	41 (29%)	
Uncertain	514 (23%)	26 (19%)	
Q31. Performing self-examination decreases mortality.			
Strongly agree	547 (24%)	42 (30%)	<0.05 *, −1222
Agreed	807 (36%)	51 (36%)	
In disagreement	154 (7%)	15 (11%)	
Strongly disagree	32 (1%)	0	
Uncertain	695 (31%)	32 (23%)	
Q32. Performing self-examination once a month helps me find lumps.			
Strongly agree	978 (44%)	65 (46%)	0.33, −0.327
Agreed	1001 (45%)	56 (40%)	
In disagreement	242 (11%)	16 (11%)	
Strongly disagree	13 (1%)	2 (1%)	
Uncertain	1 (0%)	1 (1%)	

## Data Availability

The original contributions presented in the study are included in the article; further inquiries can be directed to the corresponding author.

## Appendix A. Study Questionnaire

**Baseline Characteristics**

**Age (y)**
20–29
30–39
40–49
50–59
60–69

**Geographic Area**
North
Center
South/Islands

**Marital status**
Married
Divorced
Maiden
Separate
Widow

**Education level**
Degree
High school graduation
Junior high school diploma
Primary school
None

**Employment status**
Craftsman
Public Administration
Services/Tertiary
Student
Retired
Unemployed
**Questionnaire items**

**SECTION 1: Knowledge and beliefs about the causes and symptomatology of breast cancer**

**Q1. Do you think you are well informed about breast cancer?**
Very
Quite
Little
Not at all

**Q2. Do you think the cause of breast cancer is endocrine?**
No
Yes

**Q3. Do you think a cause of breast cancer may be previous breast disease?**
No
Yes

**Q4. Do you think a cause of breast cancer may be food?**
No
Yes

**Q5. Do you think a cause of breast cancer may be environmental factors and pollution?**
No
Yes

**Q6. Do you think a cause of breast cancer may be psychological stress?**
No
Yes

**Q7. Do you think breast pain may be a symptom of cancer?**
No
Yes
I don’t know

**Q8. Do you think the presence of a palpable nodule may be a symptom of the tumor?**
No
Yes
I don’t know

**Q9. Do you think that the change in breast shape and size may be a symptom of cancer?**
No
Yes
I don’t know

**Q10. Do you think nipple discharge may be a symptom of the tumor?**
No
Yes
I don’t know

**Q11. Do you think that nipple alteration may be a symptom of the tumor?**
No
Yes
I don’t know
**SECTION 2: Knowledge and beliefs about breast cancer prevention**

**Q12. Do you think you are well informed about breast cancer prevention?**
A lot
Quite
Little
Not at all

**Q13. What does prevention mean to you?**
Carry out periodic checks
Prevention of risk factors and early diagnosis
Prevention of complications
I don’t know

**Q14. If I found a lump, would treatment be more effective?**
Strongly agree
Agreed
In disagreement
Strongly disagree
Uncertain

**Q15. Do you consider mammography useful as an act of breast cancer prevention?**
No
Yes

**Q16. What does mammography mean to you?**
Breast self-examination and self-palpation
Ultrasound
Oncologist’s physical examination
Radiological examination of the breast
I don’t know

**Q17. At what age do you think mammography is recommended?**
<20 years old
20–30
30–40
40–50
50–60
60–70
I don’t know

**Q18. How often do you think mammography is recommended?**
Based on age/familiarity
More than once a year
Once a year
Every two years
I don’t know

**Q19. Do you consider clinical palpation useful as an act of breast cancer prevention?**
No
Yes

**Q20. Do you think bilateral ultrasound is useful as an act of breast cancer prevention?**
No
Yes

**Q21. Do you think Nuclear Magnetic Resonance Imaging (MRI) is useful as an act of breast cancer prevention?**
No
Yes

**Q22. Do you consider biopsy useful as an act of breast cancer prevention?**
No
Yes

**Q23. Do you think Computed Tomography (CT) is useful as an act of breast cancer prevention?**
No
Yes

**Q24. Do you consider blood tests useful as an act of breast cancer prevention?**
No
Yes

**Q25. Do you consider the interview with the oncologist useful as an act of breast cancer prevention?**
No
Yes

**Q26. Have you ever heard of self-examination?**
No
Yes

**Q27. In your opinion, what does self-examination consist of?**
Breast self-examination
Clinical examination of the breast (search for visible and/or palpatory findings at the breast and surrounding areas, e.g., lymphatic drainage areas, axilla, neck)
Breast radiological examination (mammography, ultrasound, MRI, biopsy, chest X-ray, scintigraphy, CT scan, PET/CT, chest X-ray)
I don’t know

**Q28. Does self-examination help in breast cancer prevention?**
Strongly agree
Agreed
In disagreement
Strongly disagree
Uncertain

**Q29. Is self-palpation not necessary if I perform a breast examination?**
Strongly agree
Agreed
In disagreement
Strongly disagree
Uncertain

**Q30. Is self-palpation not necessary if I perform periodic mammography?**
Strongly agree
Agreed
In disagreement
Strongly disagree
Uncertain

**Q31. Performing self-examination decreases mortality.**
Strongly agree
Agreed
In disagreement
Strongly disagree
Uncertain

**Q32. Performing self-examination once a month helps me find lumps.**
Strongly agree
Agreed
In disagreement
Strongly disagree
Uncertain
